# Bi-Directional Effect of Cholecystokinin Receptor-2 Overexpression on Stress-Triggered Fear Memory and Anxiety in the Mouse

**DOI:** 10.1371/journal.pone.0015999

**Published:** 2010-12-30

**Authors:** Qian Chen, Mingxi Tang, Takayoshi Mamiya, Heh-In Im, Xiaoli Xiong, Anu Joseph, Ya-Ping Tang

**Affiliations:** 1 Department of Cell Biology and Anatomy, Louisiana State University Health Sciences Center, New Orleans, Louisiana, United States of America; 2 Neuroscience Center of Excellence, Louisiana State University Health Sciences Center, New Orleans, Louisiana, United States of America; 3 Department of Pathology, Luzhou Medical College, Sichun, People's Republic of China; Institute of Cellular and Developmental Biology, Biomedical Sciences Research Center ‘Alexander Fleming’, Greece

## Abstract

Fear, an emotional response of animals to environmental stress/threats, plays an important role in initiating and driving adaptive response, by which the homeostasis in the body is maintained. Overwhelming/uncontrollable fear, however, represents a core symptom of anxiety disorders, and may disturb the homeostasis. Because to recall or imagine certain cue(s) of stress/threats is a compulsory inducer for the expression of anxiety, it is generally believed that the pathogenesis of anxiety is associated with higher attention (acquisition) selectively to stress or mal-enhanced fear memory, despite that the actual relationship between fear memory and anxiety is not yet really established. In this study, inducible forebrain-specific cholecystokinin receptor-2 transgenic (IF-CCKR-2 tg) mice, different stress paradigms, batteries of behavioral tests, and biochemical assays were used to evaluate how different CCKergic activities drive fear behavior and hormonal reaction in response to stresses with different intensities. We found that in IF-CCKR-2 tg mice, contextual fear was impaired following 1 trial of footshock, while overall fear behavior was enhanced following 36 trials of footshock, compared to their littermate controls. In contrast to a standard Yerkes-Dodson (inverted-U shaped) stress-fear relationship in control mice, a linearized stress-fear curve was observed in CCKR-2 tg mice following gradient stresses. Moreover, compared to 1 trial, 36 trials of footshock in these transgenic mice enhanced anxiety-like behavior in other behavioral tests, impaired spatial and recognition memories, and prolonged the activation of adrenocorticotropic hormone (ACTH) and glucocorticoids (CORT) following new acute stress. Taken together, these results indicate that stress may trigger two distinctive neurobehavioral systems, depending on both of the intensity of stress and the CCKergic tone in the brain. A “threshold theory” for this two-behavior system has been suggested.

## Introduction

Fear, an emotional response of animals to dangers, threats, or aversive situations that may cause bodily or mental tension, may originate from previously learned experience such as to fear a person who previously attacked you, or an innate response such as to fear an opened high place. By fearing, the body may initiate a series of reactions including strengthening the cardiovascular function, increasing the glucocorticoid release, and initiating defensive behaviors. These reactions are coordinated in order to secure a better chance for survival. In such a case, fear is situational and controllable, and will disappear when the stress no longer exists. This type of fear is functionally important, because the associated responses are adaptive, by which the homeostatic balance in the body is maintained. However, when fear becomes disproportionally intensive, chronic, and irreversible, or is not associated with any genuine risk, it represents a core symptom of anxiety. As the most common and devastating mental disorder, anxiety approximately affects 40 million adults in USA alone [1]. The excessive fears may dysregulate neuroendocrinological reactions to stress [2], cause morphological abnormality in certain brain regions [3], and eventually disable victims' mental life [4]. Apparently, anxiety brings with various maladaptive responses to the body, and consequently disturbs the homeostatic balance.

It is therefore logical to consider that the formation of fear memory and the development of anxiety should represent two distinct behavioral traits, and must undergo different molecular or/and neuronal processing. However, because memorizing certain aversive experience underlies learned fears, and at the same time, to recall certain cue(s) of stress or to imagine some potential stresses is considered at the root of many types of anxiety such as specific phobia, posttraumatic stress disorder (PTSD), and panic attack, it is generally believed that enhanced selective attention (acquisition) to stress or mal-enhanced fear memory is a neuropsychological basis for anxiety [5]. Moreover, as both fear memory and anxiety are mediated within the limbic system [6], it is difficult to identify whether this system is differentially involved in these two behavioral traits [7]. These complexities make it difficult to establish an evidence-based doctrine regarding whether or how fear memory impacts the pathogenesis of anxiety [8].

Nevertheless, evidence is emerging that the mechanism underlying fear memory and anxiety is distinctive. For example, certain genetic variants are found to associate with the onset of anxiety [9], while no evidence indicates that those identified genetic variants are able to significantly enhance fear memory *per se*. Moreover, at the behavioral level, it is generally believed that the development of overwhelming/uncontrollable fear in anxiety is positively correlated to the intensity of stress, which means that stronger stress is more anxiogenic, or has a greater effect on the expression of fear behavior in patients with anxiety. For fear memory in normal subjects, however, there is a well-established Yerkes-Dodson law [10], which states that under a mnemonic context, an inverted-U shaped relationship exists between the intensity of stress and the retention of fear [11]. In other words, fear memory will be enhanced following an increase in the intensity of stress, while when the stress is strong to certain level, it would impair, but not enhance, fear memory, and consequently, lead to a less fear response in a subject. This is also indirectly evidenced by the findings that anxiety, such as PTSD, is often accompanied by cognitive dysfunction including memory deficit [8]. Therefore, a critical puzzle for us to understand the relationship between fear memory and anxiety is that we do not know what is a pathogenic origin that could determine or switch the brain's responsiveness to stress, especially to extensive stress, from a protectively “attenuating” direction that minimizes the impact of the stress in mental processing to a detrimentally “enhancing” direction that sensitizes or exaggerates the responsiveness of the brain to stress, or even to potential stress, and consequently, leading to the onset of overwhelming/uncontrollable fear, or pathological fear, e.g. anxiety.

Recently, we have demonstrated that overexpression of the cholecystokinin receptor-2 (CCKR-2) gene in forebrain neurons significantly facilitates the development of anxiety in the mouse [12]. Virtually, the CCKergic system has long been recognized to play a critical role in anxiogenesis [13], [14]. In the CCKergic system, CCKR-2 is a predominant type of CCK receptors in the brain, and it expresses the highest level in the limbic system [15], a brain system that is essentially involved in emotion reaction [16], [17]. The majority of studies so far show that agonism and antagonism of CCKR-2 in the brain is anxiogenic and anxiolytic, respectively [18], [19]. At the same time, the CCKergic system is importantly involved in cognitive function, such as learning and memory [20]–[22]. Therefore, it is an ideal target to study how this molecule is involved in regulating fear behavior in response to stress.

In this study, we have found that the elevated CCKergic tone in the brain has a bi-directional effect on fear behavior in mice following stress, and the effect of switching from one direction to the other direction is quantitatively dependent on the intensity of stress. In addition, the interaction between the extensive stress and the CCKR-2 transgene changes the basic tone of the hypothalamus-pituitary-adrenal (HPA) axis activity, as well as its activity in response to acute stress. As a consequence, other types of hippocampus-dependent memory were impaired. Based on all these results, a two-behavior theory has been formulated and discussed.

## Results

### Inducible/reversible forebrain-specific CCKR-2 transgene in tg mice

**Figure 1:**
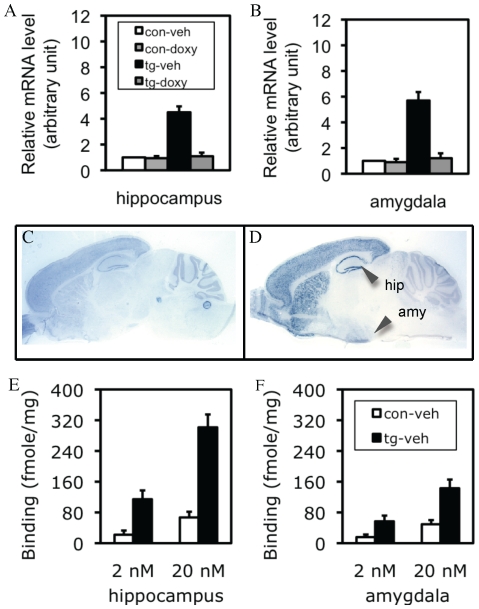
IF-CCKR-2 tg mice. (A and B) Expression level of the total CCKR-2 (endogenous and transgenic) mRNAs in the hippocampus (A) and amygdala (B) detected by real-time RT-PCR. con-veh, control mice-treated with vehicle; con-doxy, control mice-treated with doxy; tg-veh, tg mice-treated with vehicle; tg-doxy: tg mice-treated with doxy. Data are expressed as mean ± SD. (C and D) Expression pattern of the total CCKR-2 mRNAs detected by *in situ* hybridization in saggital brain sections. A moderate expression level of CCKR-2 mRNA is observed in the brain of control mice (C), while a higher expression level is observed in all the forebrain regions including the hippocampus (hip) and amygdala (amy) in tg mice (D). (E and F) CCKR binding activity in the hippocampus (E) and amygdala (F) in the presence of a low (2 nM) or a high (20 nM) dose of the ligand. Data are expressed as mean ± SD.

The transgenic constructs for Ca^+2^-calmoduline kinase-II (CaMK-II)-tTA and tetO-CCKR-2 cDNA are shown in Figure S1. The expression level of the total (endogenous and transgenic) CCKR-2 mRNAs in the hippocampus (Fig. 1A) and amygdala (Fig. 1B) of CCKR-2 transgenic (simply tg hereafter) mice-treated with vehicle was 4.5 and 5.7-fold higher than control mice-treated with vehicle, respectively. The expression of the CCKR-2 transgene could be completely inhibited by treatment with doxycycline (doxy), an analogue of tetracycline that binds to tTA to block the interaction between tTA and the tetO promoter, so that the transcription of the CCKR-2 transgene is silenced. These results were consistent with our previous results detected by Northern blots [12]. As shown in Fig. 1C, *in situ* hybridization indicated that, consistently to the published results in Allen Brain Atlas (http://mousediversity.alleninstitute.org/imageseries/show/77790627.html), the expression of CCKR-2 mRNA in the brain of control mice was mainly localized in the cortex, hippocampus, striatum, and amygdala, and a lower level in other brain regions including the thalamus/hypothalamus, and brainstem. In contrast, a higher expression level of CCKR-2 mRNAs was observed in the forebrain regions including the cerebral cortex, hippocampus, ventral striatum, caudate putamen, basal forebrain, and amygdala in tg mice (Fig. 1D). These results confirmed that the expression of the CCKR-2 transgene was both forebrain-specific and inducible/reversible.

### A higher CCKR-binding activity in the hippocampus and amygdala of tg mice

In the hippocampus, in the presence of 2 nM and 20 nM *^3^*H-CCK-8 ligand, the CCK receptor binding counts were 22.2 fmoles/mg and 67.4 fmoles/mg in control mice, and 114.8 fmoles and 301.3 fmoles/mg in tg mice, respectively (Fig. 1E), revealing a 4.5–5.2-fold difference between control and tg mice. In the amygdala, in the presence of 2 nM and 20 nM *^3^*H-CCK-8 ligand, the CCK receptor binding counts were 15.8 fmoles/mg and 49.3 fmoles/mg in control mice, and 56.8 fmoles and 142.9 fmoles/mg in tg mice, respectively (Fig. 1F), revealing a 2.9–3.6-fold difference between control and tg mice. These results indicated that the CCKR-2 transgene dramatically enhanced the CCK receptor binding activity. Our previous study showed that the doxy-treatment could completely inhibit the increased receptor binding activity in tg mice [12], so we did not repeat these experiments here again. Mice used above were al about 2–4 months old, with both female and male mice mixed.

### Impaired fear response in contextual conditioning in tg mice following 1 trial of footshock

**Figure 2:**
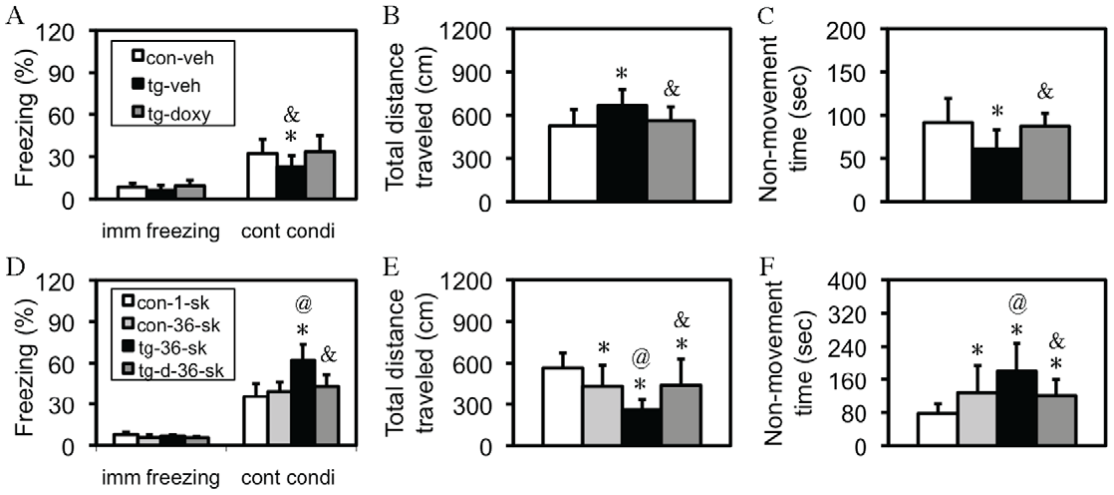
Bi-directional fear response in IF-CCKR-2 tg mice. (A–C) Fear response following 1 trial of footshock. (A) Freezing rate in immediate (imm) freezing following the US/CS coupling during training and freezing rate in contextual conditioning (cont condi) in the retention test. *, p<0.05, compared to control mice-treated with vehicle (con-veh; n = 11); ^&^, p<0.05, compared to tg mice-treated with doxy (tg-doxy; n = 10). Tg-veh indicates tg mice-treated with vehicle (n = 11). (B) Total distance traveled. *, p<0.05; ^&^, p<0.05, the same group comparisons. (C). Total non-movement time. *, p<0.05; ^&^, p<0.01, the same group comparison. All are *post-hoc* analyses following an one-way ANOVA. Data are expressed as mean ± SD. (D–F) Fear response following 36 trials of footshock. (D) Freezing rate in immediate (imm) freezing following the US/CS coupling during training and freezing rate in contextual conditioning (cont condi) in the retention test. *, p<0.001, compared to control mice-1-shock (con-1-sk; n = 11); ^@^, p<0.001, compared to control mice-36-shock (con-36-sk; n = 11); ^&^, p<0.001, compared to tg mice-36-shock (tg-36-sk; n = 11). Tg-d-36-sk indicates tg mice were treated with doxy since weaning and were stressed with 36 trials of footshock (n = 11). (E) Total distance traveled. *, p<0.05–0.001; ^@^, p<0.01; ^&^, p<0.05, the same group comparisons. (F). Total non-movement time. *, p<0.05–0.001; ^@^, p<0.05; ^&^, p<0.01, the same group comparisons. All are *post-hoc* analyses following a two-way ANOVA. Data are expressed as mean ± SD.

As shown in Fig. 2A, immediate freezing, which measured freezing response immediately following the footshock, was indistinguishable between control and tg mice, suggesting a similar level of the instinctive response to footshock. However, an one-way ANOVA revealed a significant effect of the transgene on contextual conditioning [F(2,30) = 4.52, p<0.05], and *post-hoc* analysis with Fisher's PLSD test revealed a significant group difference between tg-vehicle and either control-vehicle or tg-doxy group (p<0.05, respectively). Similar results were obtained in another type of measurement, by which a significant difference was observed in both total distance traveled [F(2,30) = 4.78, p<0.05; Fig. 2B] and total non-movement time [F(2,30) = 5.88, p<0.01; Fig. 2C]. *Post-hoc* analyses revealed a significant difference between tg-vehicle and either control-vehicle or tg-doxy group in total distance traveled (p<0.05, respectively) and non-movement time (p<0.05, p<0.01). In the cued conditioning test, neither pre-tone freezing nor cued conditioning was significantly different between these mice (data not shown). After the completion of fear-conditioning experiments, nociceptive responses were compared by measuring the current required to produce stereotype behaviors including flinching/running/moving, jumping, and vocalizing, and the results did not reveal a significant difference between any two groups of these mice (data not shown). Mice used here were about 2-4 months old, with both female and male mice mixed. A pre-statistical analysis within the same group between genders did not show a significant difference. These results indicated that the hippocampus-dependent fear memory was specifically impaired in tg mice following the mild stress.

### Enhanced fear response in tg mice following 36 trials of footshock

In order to determine whether 36 trials of footshock had a similar effect as 1 trial of footshock on fear behavior, another set of four groups of mice, as indicated in Fig. 2D, was examined with the same behavioral tests. Surprisingly, fear behavior in tg mice following 36 trials of footshock was dramatically enhanced. A two-way ANOVA revealed a significant difference in contextual conditioning for stress [F(1,20) = 6.80, p<0.05], transgene [F(1,20)  = 31.44, p<0.001], and interaction (stress X transgene) [F(1,40)  = 17.72, p<0.001], in total distance traveled for transgene [F(1,20)  = 13.06, p<0.001], and interaction [F(1,40)  = 14.48, p<0.001], but not for stress, and in non-movement time for transgene [F(1,20)  = 10.46, p<0.01], and interaction [F(1,40)  = 13.88, p<0.01], but not for stress, indicating a significant interaction between the transgene and stress in these fear responses. The results of detailed *post-hoc* analyses are marked in Fig. 2D–F, and are explained in the figure legend. Mice used here were about 2–4 months old, with both female and male mice mixed, and a pre-statistical analysis within the same group between genders did not show a significant difference. All these results, together with the result in Fig. 2A–C, indicated that the elevated CCKergic tone had a bi-directional effect, impairment or enhancement, on fear behavior, depending on the intensity of stress.

### The bi-directional effect of the CCKR-2 transgene on fear response was quantitatively dependent on the intensity of stress

**Figure 3:**
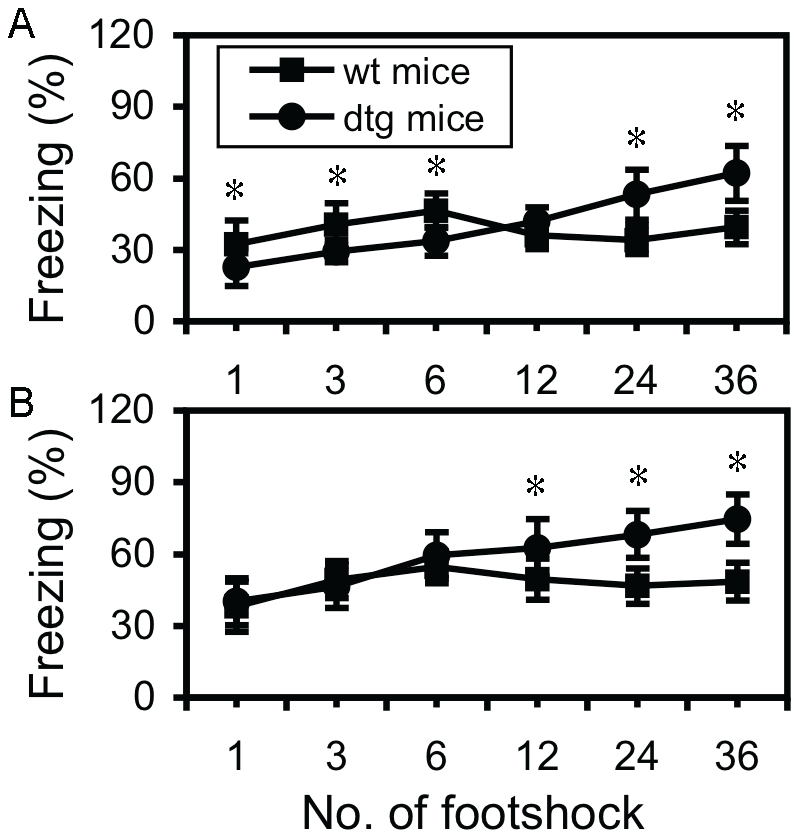
Quantitative interaction between CCKR-2 transgene and stress in fear response. (A) Freezing rate in contextual conditioning in the retention test in mice that were subjected to gradient trials of footshock in. * in 1, 3, 6, 24, and 36 trials of footshock represent p<0.05, <0.01, <0.001, <0.001, and <0.001, respectively. (B) Freezing rate in cued conditioning in the same mice. * in 12, 24, and 36 trials of footshock represent p<0.05, <0.001, and <0.001, respectively. All are Student's *t* tests. Data are expressed as mean ± SD.

In order to determine to what extent the stress could switch the fear response from one direction (impairment) to the other one (enhancement), both control and tg mice were divided into 6 groups, and were then respectively subjected to 1, 3, 6, 12, 24, and 36 trials of footshock. Both contextual conditioning and cued conditioning were examined 24 hours after the stress. In contextual conditioning, as shown in Fig. 3A, a typical inverted “U” shaped freezing curve was observed in control mice, in which a turning point from the enhancement of freezing response to the impairment was observed at 12 trials of footshock. In tg mice, however, a linearized freezing curve was observed, in which the freezing response went up following the increase of the trial number of footshock. Statistical analyses indicated that up to 6 trials of footshock, contextual conditioning in tg mice was significantly lower than that in control mice (p<0.05–0.001, respectively; Student's *t* test). Up to 12 trials of footshock, this difference disappeared, and from 24 trials up to 36 trials, a significantly higher freezing response was observed in tg mice, compared to control mice (p<0.001, respectively). In cued conditioning, a similar inverted “U” shaped freezing curve and a linearized freezing curve was respectively observed in control and tg mice (Fig. 3B). Statistical analyses revealed that the difference in cued conditioning between control and tg mice before 6 trials of footshock was not significant, indicating that the amygdala-dependent fear response was normal *per se* in tg mice. However, from 12 trials of footshock up to 36 trials of footshock, a significantly higher freezing response was observed in tg mice, compared to control mice (p<0.05–0.001; Student's *t* test). Mice used here were about 2–4 months old, with both female and male mice mixed, and a pre-statistical analysis within the same group between genders did not show a significant difference in any index examined. All these results indicated that the interactions between the CCKR-2 transgene and stress could switch fear response from one direction to another direction, and this effect was stress intensity-dependent.

### Extensive stress with 36 trials of footshock enhanced anxiety-like behavior in tg mice in open-field test

**Figure 4:**
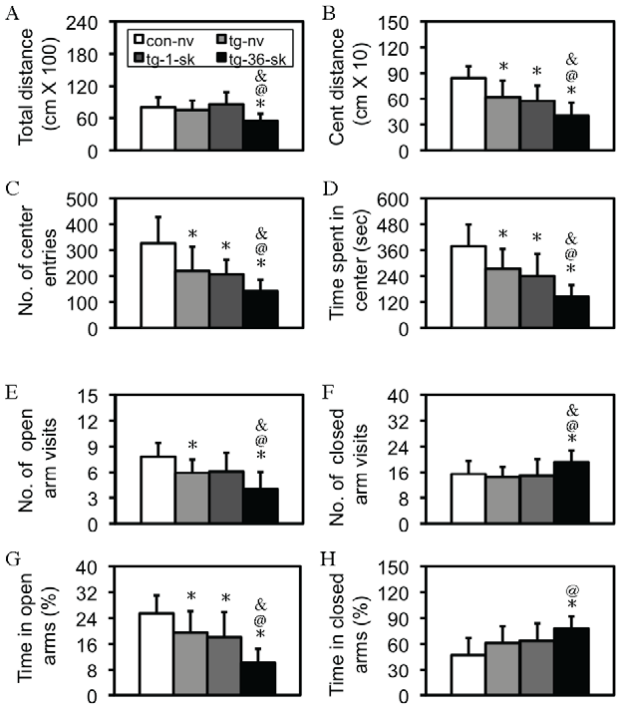
Enhanced anxiety-like behavior in tg mice following 36 trials of footshock. (A–D) Anxiety-like behavior in the open-field test. (A) Total distance traveled (total distance). *, p<0.05, compared to naïve control mice (con-nv; n = 9); ^@^, p<0.05, compared to naïve tg mice (tg-nv; n = 10); ^&^, p< 0.05, compared to tg-1-shock mice (tg-1-sk; n = 9). Tg-36-sk indicates tg mice subjected to 36 trials of footshock (n = 10). (B) Distance traveled in center area (cent distance). *, p<0.05–0.001; ^@^, p<0.05–001, ^&^, p<0.05, the same group comparisons. (C) Number of center area entries (No. of center entries). *, p<0.05–0.001; ^@^, p<0.05, ^&^, p<0.05, the same group comparisons. (D). Time spent in center area (time spent in center). *, p<0.05–0.001; ^@^, p<0.01, ^&^, p<0.05, the same group comparisons. All are post-hoc analyses following a two-way ANOVA. Data are expressed as mean ± SD. (E–H) Anxiety-like behavior in the EPM test. (E) Number of open arm visits. *, p<0.05–0.001; ^@^, p<0.05, ^&^, p<0.05, the same group comparisons. (F) Number of closed arm visits. *, p<0.05; ^@^, p<0.01, ^&^, p<0.05, the same group comparisons. (G) Time spent in open arms. *, p<0.05–0.001; ^@^, p<0.01, ^&^, p< 0.05, the same group comparisons. (H) Time spent in closed arms. *, p<0.01; ^@^, p<0.01, the same group comparisons. All are *post-hoc* analyses following a two-way ANOVA. Data are expressed as mean ± SD.

In order to study whether the increased fear response following 36-trials of footshock in tg mice was relevant to an anxiety-like behavioral phenotype, three groups of tg mice were subjected to naïve, 1 or 36 trials of footshock, respectively, and 24 hours later, these mice, together with control-naïve mice, were examined by using an open-field test. As shown in Fig. 4A–C, four indices were examined, of which the center activities are strong indices for anxious behavior, while the index of total distance traveled indicates an overall motor activity/motivation of exploratory behavior, and has some implication in anxious status under the condition that the overall center activities are significantly inhibited. Indeed, although an one-way ANOVA revealed a significant difference in total distance traveled [F(3,34)  = 4.02, p<0.05; Fig. 4A], a much higher level of significance was observed in distance traveled in center area [F(3,34)  = 11.72, p<0.001; Fig. 4B], number of center area entries [F(3,34)  = 8.57, p<0.001; Fig. 4C], and time spent in center area [F(3,34)  = 11.23, p<0.001; Fig. 4D] was observed. Detailed *post-hoc* analyses, as showed in Fig. 4A–D, revealed that (1) the CCKR-2 transgene was anxiogenic. Although no significant difference was found in total distance traveled between control and tg mice (Fig. 4A), this index might be more relevant to a general motor activity. (2) An interaction between the transgene and extensive, but not mild, stress in the anxiogenesis was observed, since a significant difference was observed in tg-36-shock mice in all indices examined, compared to any other group of mice. Mice used here were about 2–4 months old, with both female and male mice mixed, and a pre-statistical analysis within the same group between genders did not show a significant difference in any index examined.

### Extensive stress with 36 trials of footshock enhanced anxiety-like behavior in tg mice in EPM test

To further confirm whether anxiety-like behavior was enhanced following the extensive stress, another set of mice was examined with an EPM 24 hours after the stress of 36 trials of footshock. An one-way ANOVA revealed a significant difference in number of open arm visits [F(3,38)  = 7.52, p<0.001; Fig 4E], number of closed arm visits [F(3,38)  = 2.94, p<0.05; Fig. 4F], time spent in open arms [F(3,38)  = 11.54, p<0.001; Fig. 4G], and time spent in closed arms [F(3,38)  = 4.96, p<0.01; Fig. 4H]. Detailed *post-hoc* analyses, as showed in Fig. 4E–H, revealed very similar results as those in open-field test. Mice used here were about 2–4 months old, with both female and male mice mixed, and a pre-statistical analysis within the same group between genders did not show a significant difference.

### Extensive stress with 36 trials of footshock impaired spatial learning and memory in tg mice in Morris water maze test

**Figure 5:**
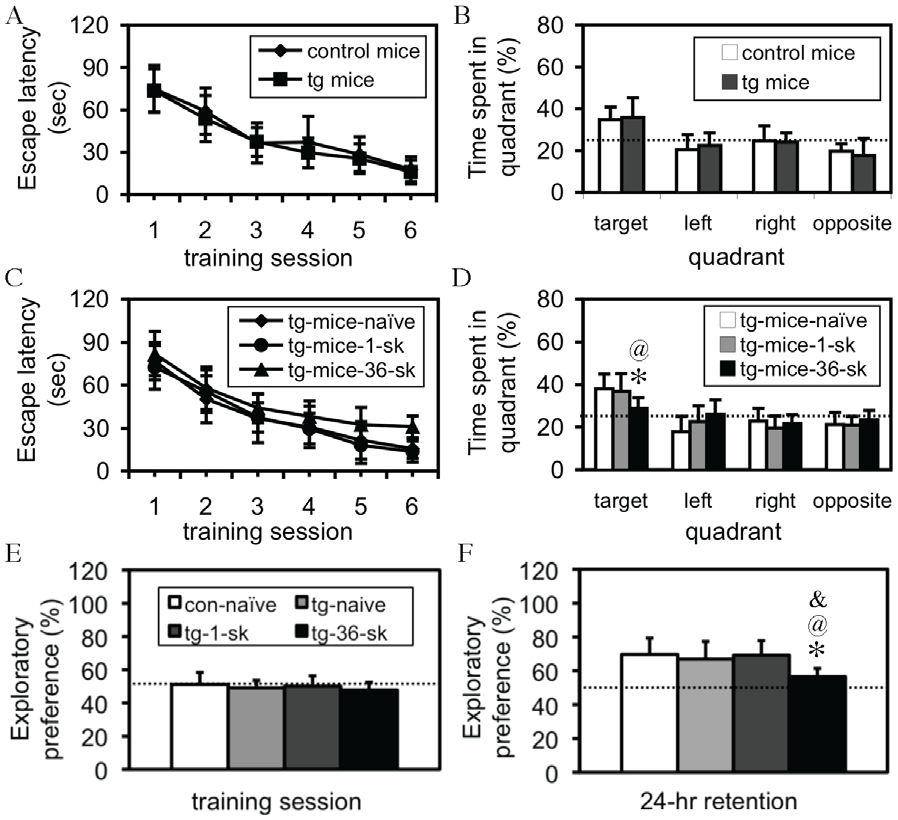
Impaired hippocampus-dependent memories in tg mice following 36 trials of footshock. (A–D) Spatial learning and memory in the Morris water maze test. (A) Learning curves for both control (n = 10) and tg (n = 10) mice. Statistical analysis of the escape latency does not show a significant difference between these two groups. (B) A probe test does not reveal a significant difference between these mice either. Dotted line represents the performance at the chance level (25%). (C) Learning curves for tg-naïve (n = 8), tg-1-shock (sk; n = 11) mice, and tg-36-shock (sk; n  = 10) mice. A significant interaction between the CCKR-2 transgene and extensive stress is observed in escape latency. (D) A probe test also reveals a significant difference in time spent in the targeting quadrant. Dotted line represents the performance at the chance level (25%). *, p<0.05, compared to naïve tg mice; ^@^, p<0.05, compared to tg-1-shock mice; All are Student's t tests. Data are expressed as mean ± SD. (E and F) Recognition memory in the novel object recognition test. (E) Exploratory preference in the training session. There is no significant difference in exploratory preference between these groups. Dotted line represents the performance at chance (50%). (F) Exploratory preference in the retention test. Dotted line represents the performance at chance (50%). *, p<0.01, compared to naïve control mice (con-naive; n = 10); ^@^, p<0.05, compared to tg-naïve mice (tg-naïve; n = 10); ^&^, p<0.01, compared to tg-1-shock mice (tg-1-sk; n = 8). All are analyzed with a Student's *t* test. Data are expressed as mean ± SD.

We first examined whether the transgene itself affected spatial learning and memory. As shown in Fig. 5A, the escape latency in either control or tg mice dramatically decreased following training, and a within-group one-way ANOVA revealed a highly significant difference in both control [F(5,54)  = 24.82, p<0.001] and tg mice [F(5,54)  = 18.67, p<0.001], indicating that all these mice were able to learn the task. Similarly, statistical analyses did not reveal a significant difference in either the escape latency during all the training sessions (repeated ANOVA) or in time spent in the targeting quadrant during a probe test (Student's *t* test) between control and tg mice (Fig. 5B), indicating that spatial learning and memory was basically intact in tg mice. We then examined whether stress affected spatial learning and memory in tg mice. To do so, three groups of tg mice were subjected to naïve-shock (mice were individually put into the shock box, but without shock), 1 trial of shock, and 36 trials of shock, respectively, and 24 hours following the stress, mice were trained in the water-maze. All these mice learned the task, as a within-group one-way ANOVA revealed a significant effect of training on escape latency in tg-naïve [F(5,42)  = 17.67, p<0.001], tg-1-shock [F(5,60)  = 21.61, p<0.001], and tg-36-shock mice [F(5,48)  = 9.08, p<0.01]. However, a repeated ANOVA revealed a significant effect of the stress and transgene on escape latency [F(2,25)  = 6.43, p<0.01], and *post-hoc* tests further revealed difference between groups (Fig. 5C). A probe test showed a similar result (Fig. 5D). Mice used here were about 2–4 months old, with both female and male mice mixed, and a pre-statistical analysis within the same group between genders did not show a significant difference. These results indicated that the interaction between the transgene and extensive, but not mild, stress impaired spatial learning and memory.

### Extensive stress with 36 trials of footshock impaired recognition memory in tg mice in novel object recognition test

To determine whether the memory deficit in tg mice following the extensive stress was a general amnesic effect of the anxiogenesis, another hippocampus-dependent test, novel object recognition test, was employed. The difference in the amount of time spent on exploring, or exploratory preference for, any object during the training session was not significant (Fig. 5E), indicating that all these mice had a similar curiosity or motivation to explore these objects. In a retention test, however, an one-way ANOVA revealed a significant effect of the transgene and extensive stress on the task performance [F(3,33)  = 3.48, p<0.05], and *post-hoc* analyses showed a significant difference in exploratory preference between tg-36-shock mice and any other group of mice (p<0.05–0.01; Fig. 5F), indicating that the extensive stress, but not mild stress, impaired recognition memory in tg mice. Mice used here were about 2–4 months old, with both female and male mice mixed, and a pre-statistical analysis within the same group between genders did not show a significant difference.

### Extensive stress with 36 trials of footshock prolonged the activation of HPA axis activity

**Figure 6:**
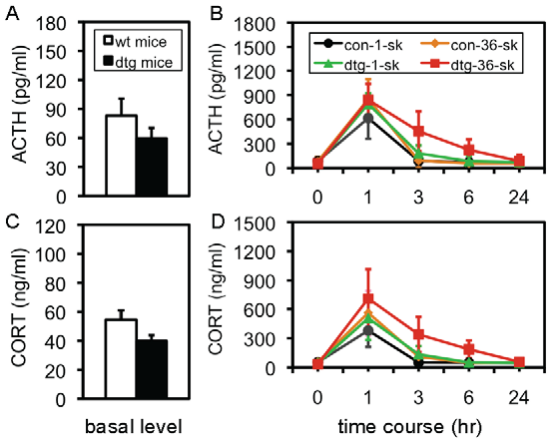
Increased HPA axis activity in tg mice following 36 trials of footshock. (A) Basal serum level of ACTH in naïve control mice (con-naïve) and naïve tg mice (tg-naïve). A tendency of a lower level is shown, but it is not significant. Data are expressed as mean ± SEM. (B) Time-course of ACTH response following 1 or 36 trials of footshock. Control-1-shock mice (con-1-sk), control-36-shock mice (con-36-sk), tg-1-shock mice (tg-1-sh), and tg-36-shock mice (tg-36-sk) were examined. A repeated ANOVA showed: A: F(3,34)  = 11.79; B: F(4,136)  = 231.16; A×B: F(3,12)  = 5.124, all with p<0.001, indicating a robust interaction. Detailed *post-hoc* analyses are described in the text. (C) Basal serum level of CORT in naïve control mice (con-naïve) and naïve tg mice (tg-naïve). A tendency of a lower level is shown, but it is not significant. Data are expressed as mean ± SEM. (D) Time-course of CORT response following stresses. The same groups of mice above were examined. A repeated ANOVA showed: A: F(3,34)  = 7.33, p<0.001; B: F(4,136)  = 5.16, p<0.001; A×B: F(3,12)  = 2.663, p<0.01, indicating a robust interaction. Detailed *post-hoc* analyses are described in the text.

Given that the HPA axis system is dynamically involved in various fear responses [23], and that there are robust interactions between the HPA axis system and CCKergic system [24]–[26], it is critical to study how the HPA axis system was involved in this transgene/stress co-mediated fear behavior. Both adrenocorticotropic hormone (ACTH) and glucocorticoids (CORT) were examined by a time-course of 0 (naïve), 1, 3, 6, and 24 hours following a single or 36 trials of footshock. Data from two control-naïve and two tg-naïve subgroups were respectively pooled together to be considered as the basal level in control and tg mice, respectively. As shown in Fig. 6A and C, although the difference between control-naive (n = 20) and tg-naïve (n = 20) mice in ACTH (83.1 vs. 59.6 pg/ml; p = 0.0735) or CORT (54.5 vs. 39.9 ng/ml; p = 0.0620) did not reach a significant level, a tendency of a lower basal level of the HPA axis activity in tg mice was indicative. Following stresses, a repeated ANOVA revealed a significant effect of the transgene and stresses on ACTH [F(3,12)  = 5.12, p<0.001] and CORT [F(3,12)  = 2.66, p<0.01]. Detailed post-hoc analyses are as below: following either the mild or extensive stress, both ACTH and CORT reached the peak level at 1 hour in control and tg mice, and a significant difference (p<0.05) in both hormones was found between control-1-shock (n = 11) and control-36-shock mice (n = 11), indicating that the peak level was directly related to the intensity of the stress in control mice. This difference was not observed between tg-1-shock (n = 9) and tg-36-shock mice (n = 10), as the peak level of both ACTH and CORT in tg-1-shock mice was up-regulated to some extent, compared to control-1-shock mice (p = 0.065 and p = 0.093, respectively). Moreover, the difference in the peak level of either hormone between control-36-shock and tg-36-shock mice was not significant, indicating that there might be a ceiling effect of the transgene and extensive stress on the HPAA activity. In control mice, the increased ACTH returned to the basal level within 3 hours after either stress, but a higher CORT was still observed between control-1-shock (n = 10) and control-36-shock mice (n = 11) at this time-point (p<0.05), indicating that the CORT response lasted longer following the extensive stress. In tg mice, the increased activity was dramatically extended, especially following the extensive stress. A significant difference in ACTH was found between control-1-shock (n = 10) and tg-1-shock mice (n = 10) at 3-hour (p<0.05), between control-36-shock (n = 11) and tg-36-shock mice (n = 10) at 3-hour (p<0.01), and between control-36-shock (n = 11) and tg-36-shock mice (n = 9) at 6-hour (p<0.01); and a significant difference in CORT between control-1-shock and tg-1-shock mice at 3-hour (p<0.05), between control-36-shock and tg-36-shock mice at 3-hour (p<0.05) and at 6-hour (p<0.05). Overall, these results indicated that both the peak level of the HPA axis activity and time window of the activation were directly related to the intensity of stress, and that although the transgene did not increase the peak level, it significantly extended the activation time of both ACTH and CORT. Mice used here were about 2–4 months old. In order to avoid a potential gender effect, only male mice were used in these experiments.

## Discussion

In this study, we have explored the relationship between fear memory and anxiety by using our previously engineered IF-CCKR-2 tg mice, together with different stress paradigms. We have identified a molecular substrate that could change the responsiveness of the brain to stress from a self-regulatory way to an uncontrollable pathogenic way to fear the stress. This study has provided valuable evidence to indicate that fear memory and anxiety represent two distinct neurobehavioral systems in the brain, and the factors that could switch these systems include the intensity of stress and the endogenous CCKergic activity.

First of all, there were several unique merits in the approaches used in this study. The inducible overexpression ensures that the phenotypes observed in tg mice are due to the transgene expression, but not any other non-specific effects such as those that may be associated with transgene genomic insertion. For example, the results that behavioral responses in tg-36-shock mice-treated with doxy returned to control-36-shock level confirmed this notion (Fig. 2). Based on this result, together with the results in receptor binding assay (Fig. 1A and B), it was not needed to examine the doxy effect in every experiment thereafter. The forebrain-specific overexpression does not only minimize certain possible non-specific effects, but also provides an ideal model for the use of a fear-conditioning paradigm, which detects hippocampus-dependent and -independent fear memory simultaneously [27]. With these advantages, the first important finding is that in tg mice, only contextual (hippocampus-dependent), but not cued (hippocampus-independent), conditioning was impaired following the mild stress (1 trial of footshock). The results from the measurements of total distance traveled and non-movement time, both of which were highly compatible to the results of freezing response (Fig. 2), and the demonstration of the same nociceptive sensitivity to footshock between control and tg mice, further consolidate this observation. Given that the amygdala plays a central role in fear response [16], these observations indicate that the CCKR-2 transgene does not impair amygdala-mediated fear response *per se*, but only impairs the hippocampus-encoded fear memory. This differential effect provides a basis for a further study of the relationship between stress, fear memory, and anxiety, in this study.

Another finding is that other types of hippocampus-dependent memory in tg mice were intact (Fig. 5). Since the first discovery of the memory deficit in so-called H.M. patient [28], the role of the hippocampus in learning and memory has been well established, primarily based on lesion studies [29]. For example, the uses of pharmacological, neurosurgical, and genetic devices to lesion the hippocampus have revealed the essential role of the hippocampus in multiple types of memory including spatial, emotional, and recognition memory [30]–[33]. In contrast to those lesion studies, in which the information transduction in the neural chain within the hippocampus was blocked, the expression of the CCKR-2 transgene in the mouse would unlikely produce a similar “lesion” to the hippocampus, and thus the effect on memory would be more specific. At the same time, this specific effect also suggests that different memories may have a different molecular basis. Indeed, there is abundant evidence that supports this notion [34]. For example, in c-fos null mutant mice, both spatial and contextual conditioning memories were impaired. When another c-fos family member, Fra-1, was knocked-in into the c-fos locus to replace the c-fos null allele, contextual fear memory, but not spatial memory, was rescued [35].

The most important finding in this study is that different intensities of stress could trigger two directional, impairment and enhancement, fear responses in tg mice. First, in response to mild stress, contextual fear memory in tg mice, in comparison with control mice, is impaired, which was evidenced by a lower freezing response (Fig. 1A), together with a lower level of other fear behavior (Fig. 2B and C). Second, the significant difference between control-1-shook and wt-36-shock groups in total distance traveled (Fig. 1E) and non-movement time (Fig. 1F), but not in freezing response (Fig. 1D), indicates that the extensive stress has certain, but not consistent, effect on these fear responses. At the same time, the significant difference between control-36-shock and tg-36-shock groups in all the indices (Fig. 1D–F) suggests a strong effect of the CCKR-2 transgene and an interaction between stress and the transgene. The consistently significant difference between control-1-shock and tg-36-shock groups in all the indices examined (p<0.001) further confirmed this interaction. Moreover, this interaction is also evident in other tests. As showed in Fig. 4E–H, compared to control-naïve mice, a significant difference was only observed in some, but not all, indices, indicating that anxiety-like behavior in tg mice in EPM was not robust as that in open-field test. Following the extensive stress, however, tg mice exhibited more consistent and robust anxiety-like behavior in all the indices examined when compared to control-naïve mice, and a significant difference in all the indices between tg-naïve and tg-36-shock mice, and a difference in most indices between tg-1-shock and tg-36-shock tg mice. No significant difference was found in any index between tg-naive and tg-1-shock mice. These results do not only confirm the interaction, but also reveal that this interaction depends on the intensity of the stress. It should be noted that in both open-field and EPM tests we did not examine control mice with stress (e.g. 1 vs. 36 trials of shock), because 1) these experiments aimed on the bi-directional effect of the transgene on fear response and 2) this bi-directional effect was not observed in control mice. Most importantly, in response to extensive stress, although control mice showed only a slight increase in contextual freezing (Fig. 2D), a significantly higher level of other fear behavior was observed (Fig. 2E and F). In contrast, in tg mice, all these fear behaviors were significantly enhanced following the extensive stress. Since the enhanced anxiety-like behavior (Fig. 4), together with the impaired memory (Fig. 5), was also observed in tg mice following the extensive stress in other behavioral paradigms, it is reasonable to consider that the enhanced fear response in tg mice is not due to enhanced fear memory by a sensitization mechanism. Therefore, it seems clear that the expression of the CCKR-2 transgene makes this two-directional fear response observable: 1 trial of footshock, which typically produces fear memory [36], [37], impairs fear memory in tg mice, while 36 trials of footshock, which represent anxiety-like behavior-causable stress to the mouse [37], [38], dramatically strengthens fear response in tg mice, and other anxiety-like behaviors (Fig. 4). These results thus indicate that there are two neurobehavioral systems in the brain, fear memory and anxiety, and the expression of the CCKR-2 transgene is able to probe these systems in the mouse.

Another important effort in this study is that we tried to elucidate a quantitative relationship between the intensity of the stress and the CCKR-2 transgene in fear behavior. As shown in Fig. 3, in control mice, both contextual freezing and cued freezing go up following the increase in the intensity of the stress up to certain level, and then go down following further increase of the intensity. However, at the most intensive stress used here (36 trials), the freezing in both conditionings does not decrease further, but slightly increases instead. Thus this stress/freezing curve in control mice represents a non-typical inverted-U shaped curve for both conditionings. As regarding why the 36-trial of footshock did not further decrease the contextual conditioning in control mice, it might be related to a mixture phenotype of impaired fear memory (decrease) and enhanced anxiety (increase), because extensive stress might be both anxiogenic and amnesic. Most distinguishably, a linearized stress/freezing curve was observed in tg mice in both contextual freezing and cued freezing, although a higher slop was observed in contextual conditioning, due to the impairment at the lower number trials of footshock. Based on the well-established Yerkes-Dodson law as described above, our results are insightful at the following aspects. First, there is indeed a turning point, from enhancement to impairment, for fear behavior in response to stress in animals for fear memory formation. Second, a higher CCKergic tone in the brain disables this turning point, or the Yerkes-Dodson law [11], and thus, leads to a super-level of fear behavior, which phenotypically resembles to anxiety in the humans. Third, at the phenotypical level, an observed fear behavior may be a mixed phenotype. An increased fear response may be due to enhanced fear memory or enhanced anxiety-like behavior, a decreased fear response may be due to impaired fear memory or less anxiety, while a minor change or non-change in fear response, such as in 36 trials-triggered fear response in current study, may be due to a mixture of impaired fear memory and enhanced anxiety-like behavior. Thus, it is questionable to use a single fear paradigm for the evaluation of anxiety-like behavior in the rodents. A combination of other behavioral tests, as described in Fig. 4, is critically needed. This may explain why so many inconsistent findings, especially for the role of the CCKergic system and many other neurochemical systems in memory and anxiety, were reported. Without a definition of which neurobehavioral system is activated in a system, it may be not sufficient enough to make a conclusion that either fear memory or anxiety is enhanced or attenuated. Thus, our results for the first time put forward this new concept regarding how to evaluate fear behavior in the rodents, in terms of fear memory and anxiety. We believe that this new concept will have a significant impact in the field.

**Figure 7:**
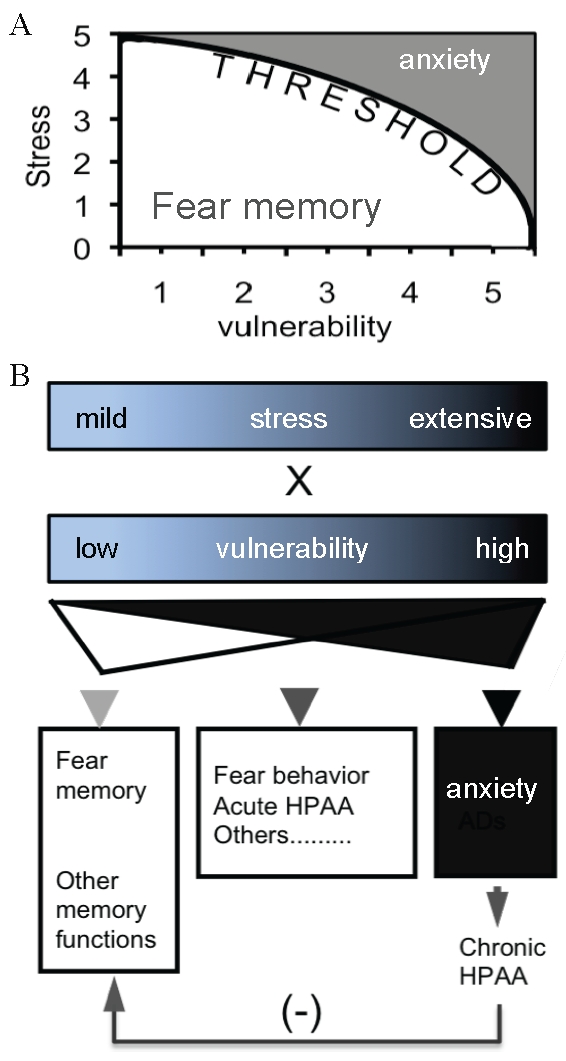
A “threshold theory” for two-behavior system. (A) The expression of a fear phenotype is dependent on both the intensity of stress and degree of vulnerability. There is a linear relationship between these two factors in the pathogenesis of anxiety. (B) The direction of the development of fear behavior is dependent on the integrative force of the interactions between stress and vulnerability. A lower force leads to fear memory, which is accompanied by many adaptive responses such as acute HPA axis response. An extremely higher force leads to anxiety or anxiety-like behavior, which is accompanied by many maladaptive responses including chronic activation of the HPA axis system and many others.

Although from the current study we still do not know how the CCKR-2 transgene differentially regulates these two types of fear response, an important clue has emerged from our HPA study, by which we obtained three important findings. First, the basal level of the HPA axis activity in tg mice was lower, compared to control mice, even though the difference was not significant (Fig. 6). Second, in control mice, the peak level of the HPA axis activity was correlated to the intensity of the stress, while in tg mice, this correlation disappeared, indicating that the sensitivity to stress in tg mice increased. Third, the decay time of the HPA axis activity was dramatically prolonged in tg mice following the extensive stress (Fig. 6B and D), despite that the basal level in tg mice was lower. It has been well established that previous chronic stress in the animals down-regulates the HPA axis activity, but enhances its response to a novel acute stress, despite the negative feedback effects [39]. In addition, robust evidence reveals a significant interaction between the CCKergic and HPA axis systems [24]–[26]. Accordingly, the elevated CCKergic tone in the brain may mimic the effect of chronic stress by working as a “constitutive intrinsic molecular/neuronal stressor” for the animals, and thus to make the mice more sensitive to stress. Taken into account of all these findings, a “threshold theory” is suggested. As shown in Fig. 7A, both the intensity of stress and the vulnerability of a subject to stress determine the development of fear phenotype, and there is a linearized relationship between these two factors in the pathogenesis of anxiety. In most cases, because the integrative force of the interactions between stress and vulnerability does not reach to the threshold for a pathogenesis of anxiety, the direction leads to the formation of fear memory, which is featured by many adaptive responses such as an acute HPA axis response and many others (Fig. 7B). These responses are favorable for memory formation [40], [41]. In the case of extensive stress or a higher vulnerability, the integrative force of the interactions reaches over the threshold, and thus the direction is leading to the pathogenesis of anxiety, which consequently produce maladaptive responses including a chronic HPA axis activity and many others. These maladaptive responses may damage brain structures including the hippocampus, and in turn impair learning and memory [42]. Both the increased HPA axis activity and impaired memory were observed in our tg mice. Obviously, the elevated CCKergic tone, in the case of our study, and many other factors in the cases of other studies, exert the anxiogenic effect(s) by increasing the vulnerability to stress.

It is not clear how the elevated CCKergic tone contributes to a higher vulnerability. Apparently, the interaction with the HPA axis system is not the only mechanism, either for the enhanced anxiety-like behavior or impaired memory. The activation of CCKR is associated with Ca2+ release, PKC activation, stimulation of PLA2, and cAMP production [43]. It might be possible that these downstream molecules play a part in regulating this two-behavior system. For example, a competitive interaction between long-term depression (LTD) and long-term potentiation (LTP) in the hippocampus may underlie the storage of emotional memory and stress-induced amnesia [44], [45]. Extensive stress may reverse LTP, which was produced by an emotional episode, to LTD, so that the synaptic plasticity in the hippocampus is disturbed [44]. The switching from LTP to LTD may lead to both impaired emotional memory and enhanced anxiety [46], [47]. As all these signal pathways are involved in these forms of synaptic plasticity, it will be interesting to further study how the environmental stress changes the synaptic plasticity in the hippocampus. Recently, CCK was found to excite interneurons in the amygdala, and thus, the CCKergic system may change fear behavior in response to stress [48]. Indeed, robust evidence shows that the CCKergic system is dynamically involved in response to stress. For example, following stress including acute stress [49], chronic stress [50], early-life stress [51], or social isolation [52], the CCKergic function was significantly enhanced. Moreover, CCKR-2 agonists could only produce, or produce more pronounced, anxiogenic effect in stressed animals, but not in un-stressed animals [53], and patients with anxiety were more sensitive to CCKR-2 agonists than normal controls [54]. Meanwhile, the expression of anxiety was correlated with increased CCKergic tone [55], [56]. At the same time, accumulating evidence indicates that either the CCKergic system or the specific CCKR-2 receptor is significantly involved in genetic vulnerability to anxiety [57], [58] including PTSD [59], depression [60], [61], and schizophrenia [62], [63]. Therefore, it is evident that a change in the CCKergic system, especially the CCKR-2, may play an essential role in regulating stress-related behavior.

In conclusion, the results presented here have for the first time provided experimental evidence that stress may trigger two different neurobehavioral systems in the brain, depending on the intensity of the stress and the endogenous CCKergic tone. These results also reveal that the development of fear memory and anxiety-like behavior do not share the same molecular/neuronal mechanism. It is needed to further investigate, however, how the CCKergic system drives this two-behavior system in our future studies.

## Materials and Methods

### IF-CCKR-2 tg mice

All experimental procedures for this study for the use of animals were previously reviewed and approved by the institutional animal care and use committee (IACUC) at the Louisiana State University Heath Sciences Center at New Orleans (ID No. 2654), and all of the experiments were conducted in accordance with the Guide for the Care and Use of Laboratory Animals published by the US National Institutes of Health. A tTA/tetO system (pTet-off; Clontech) was used to produce IF-CCKR-2 tg mice [12]. Briefly, two single transgenic mouse strains, ∂-Ca^2+^-calmodulin kinase II (CaMK-II)-tTA and tetO-CCKR-2, were needed. The tTA was flanked by an upstream 0.6 kb splicing signal (p265) and a downstream 0.5 kb SV-40 poly-A signal (p265). A CCKR-2 cDNA (1.3 kb), which was amplified by RT-PCR with the total RNA extracted from the brain of a male B6/CBA F_1_ mouse (Jackson Laboratory) with the primers of 5′-CGG GAT CCA TGG ATC TGC TCA AGC TG-3′ and 5′-GCT CTA GAT CAG CCA GGT CCC AGC GT-3′, was flanked by an upstream 0.6 kb splicing signal (p265) and a downstream 1.1 kb β-globin poly-A signal, and was sub-cloned into a pTRE2 vector (Clontech). The cassettes were separately injected into the pro-nucleoli of B6/CBA F_1_ zygotes. Founders and their transgene copy numbers were determined by Southern blot. Founders with a suitable transgene copy number were crossed into B6/CBA F_1_ mice to produce single transgenic mice, and then to produce hemizygous double transgenic mice by breeding these two single transgenic mice together. The genotypes were determined by PCR amplification of the tTA (5′-AGG CTT GAG ATC TGG CCA TAC-3′ and 5′-AGG AAA AGT GAG TAT GGT G-3′) and CCKR-2 transgene (5′-ACG GTG GGA GGC CTA TAT AA-3′ and 5′-GAG TGT GAA GGG CATG CAA-3′) with genomic DNA from tails. Tg mice used here were around 14–20 generations since they were generated, during which tg mice were backcrossed into B6/CBA F_1_ (Jackson Laboratory) mice in every 5–6 generations, in order to avoid an inbreed effect. Single transgenic (either tTA or tetO-CCKR-2 only) and wild-type littermates of tg mice were used as controls. Mice were kept in standard mouse cages under a standard condition (12 h light/dark cycle, temperature at 22°C, and humility at 75%) with food and water *ad libitum*. Mice used in this study were about 2–4 months old.

### Real-time RT-PCR and *in situ* hybridization

We used real-time RT-PCR and *in situ* hybridization to detect the expression level and pattern of the total CCKR-2 mRNAs (endogenous and transgenic mRNAs) in the brain. For real-time RT-PCR, total RNA was extracted from the hippocampus and amygdala with Trizol (Invitrogen), and was purified by RNEasy columns (Qiagen). Reverse transcription (RT) was performed by using a SuperScript® III First-Strand synthesis system (Invitrogen). A fluoresce probe recognizing both the endogenous and transgenic CCKR-2 mRNAs was used with a 40-cycle of PCR amplification (Applied Biosystem, 7900^th^). The expression level was normalized by the 18S rRNA expression. Experiments were repeated three times with three individual mice. For *in situ* hybridization, both control and tg mice were deeply anesthetized with sodium pentobarbital (Sigma-Aldrich; 50 mg/kg, i.p.), and were perfused transcardially with 1 X PBS followed by 4% paraformaldehyde (PFA). Brains were then post-fixed overnight with 4% PFA in 20% sucrose. Sagittal sections (30 µm) were made with a Cryostat (Leica, CM 1900). A cRNA probe that could not distinguish the endogenous and transgenic CCKR-2 mRNAs was obtained from an *in vitro* transcription and was labeled with a digoxigenin labeling kit (Roche). The procedures of the hybridization followed the instruction of the provider. The hybridization signal was visualized by BCIP and analyzed with an Olympus microscope (SZ-PT) and the Q-imaging system.

### CCKR binding assay

CCKR-binding assays were conducted with ^3^H-CCK-8 (propiony-^3^H-sulfated and propionylated CCK-8, 93.0 Ci/mmol; Amersham Pharmacia Radiochemicals) to determine the CCKR binding activity in both the hippocampus and amygdala as described previously [12]. Because we have already confirmed the saturated binding curve in the forebrain of tg mice [12], we did not repeat these measurements here. Instead, only two concentrations of ^3^H-CCK-8, 2 and 20 nM, were examined in each sample with triplicate measurements. Hippocampi or amygdalae from 3 mice were pooled together, respectively, and the experiments were repeated three times (so total 9 mice in each group). Nonspecific binding was determined by using 1 µM cold CCK-8 under the same incubation conditions above. The specific binding was calculated by the total binding (cpm) - nonspecific binding (cpm).

### Mild stress and fear behavior

Mild stress was delivered by one trial of footshock with a fear-conditioning paradigm (Coulbourn Instruments) as described previously [64]. Briefly, mice were individually put into a shock chamber and were allowed to freely explore the chamber for 2.5 minutes. A tone at 90 dB and 2,800 Hz (conditioned stimulus; CS) was then delivered for 30 seconds, and at the last 2 seconds, footshock at 0.6 mA (unconditioned stimulus; US) was delivered for 2 seconds. After this CS/US pairing, mouse was allowed to stay in the chamber for another 30 seconds. Fear behavior was measured 24 hours after the CS/US pairing. In contextual conditioning, mice were individually put back the same chamber where they received the shock, and freezing behavior was recorded for 3 minutes by using a 5-second sampling method by an experimenter who was blind to mouse genotypes. In order to have a relatively completive dada analysis, both total distance traveled and total non-movement time were automatically recorded by the photo-beam scanning system during the contextual conditioning. After the contextual conditioning, cued conditioning was examined, during which mice were individually put into a novel chamber (different in floor, wall, and the shape), and were allowed to freely explore the chamber for 3 minutes (pre-tone stage). The same tone used in the training was then delivered for 3 minutes (cued conditioning). Freezing behavior was recorded by using the same 5-second sampling method. Due to both a technical difficulty and the high comparability between the freezing and total distance traveled/non-movement time from the contextual conditioning, we did not measure total distance traveled/non-movement time in cued conditioning. The freezing rate was calculated as freezing sampling number/total sampling number X 100%.

### Extensive stress and fear behavior

Extensive stress was delivered by 36 trials of footshock by using the same shock chamber as described above, while the procedures were different. Briefly, mice were individually put into the shock chamber and were allowed to freely explore the chamber for 2.5 minutes, and then mice received footshock at 0.6 mA for 36 times (trials) in a period of 6 minutes, during which an interval of 10 seconds was set between trials, and each trial lasted for 1 second. After the completion of these 36 trials of footshock, mice were allowed to stay in the chamber for another 30 seconds, and then were returned to their homecages. Fear behavior was examined 24 hours after the stress by putting them individually back to the same chamber where they received the footshock. As the same as above, freezing response, and total distance traveled/total non-movement time were respectively recoded by the 5-second sampling method and photobeam scanning system for a period of 6 minutes.

### Quantitative studies of the effect of stress on fear behavior

Both control and tg mice were divided into 6 groups, which received 1, 3, 6, 12, 24, and 36 trials of footshock respectively. The same shock chamber as described above, was used, while the procedures were different. Briefly, mice were individually put into the shock chamber and were allowed to freely explore the chamber for 2.5 minutes, and then mice received footshock at 0.6 mA for 1 second for different times (trials) as scheduled. In each trial of footshock, a tone at 90 dB and 2,800 Hz was delivered for 5 seconds, and at the last second of the tone, the shock was delivered. So, 1, 3, 6, 12, 24, and 36 trials of footshock were completed within 10, 30, 60, 120, 240, and 360 seconds, respectively, in addition to the pre-CS/US coupling stage (2.5 minutes) in every group of mice. Mice were returned to their homecage immediately after the stress, and fear behavior, as described above, were examined 24 hours later.

### Open-field test

Open-field behaviors were examined by using an automatic-recording open-field working station (MED Associates) as described previously [12]. The open-field box (40×40×30 cm high) was divided into 16 identical squares by invisible but computer-detectable lines, and the open-field was illuminated by a dim light (20 lux). Two sets of 16 pulse-modulated infrared photobeam were placed on opposite walls 2.5 cm apart from the wall to record X-Y ambulatory movements. Exploratory behavior in the box was computer-interfaced at a sampling rate of 100-ms resolution. Mice were transported to the behavioral room to adapt the environment for at least 1 hour before the experiment. Behavioral indices including total distance traveled, ambulation counts, and number of rearing were recorded automatically by the scanning system for 60 minutes.

### Elevated-plus maze (EPM) test

The apparatus (Med-Associates) of the EPM consisted of a platform (7×7 cm) and four dark gray Plexiglas arms, of which two were open arms (67×7 cm) and two were closed arms (67×7×17 cm). The open arms and closed arms formed a cross shape with the two open arms opposite each other and so the two closed arms opposite each other too. The maze was set at 55 cm above the floor and was dimly illuminated (20 lux). Photobeam cells (connected to a computer), placed at two different directions along length of each arm, allowed detecting the passage of the animal from the central platform to any arm. A video tracking system (EthoVision) was placed above the apparatus to record behavioral responses, and data were automatically analyzed by the tracking-system. During testing, mice were individually placed in the center of the platform by facing to a closed arm, and were allowed to freely explore the maze for 5 minutes. Number of open arm and closed arm visits, and time spent in open arms and closed arms were separately recorded.

### Morris water-maze test

A Morris water maze was used to evaluate spatial learning and memory as described previously [64]. Briefly, a circular water tank (diameter 100 cm and 75 cm in high) was filled with water that was made opaque with non-toxic white paint (Reeves &Poole group, Toronto, Canada) by 3/4 of the tank. The water tank was surrounded by a black curtain 1 meter away, with three visible signs on the curtain. A round platform (diameter 15 cm), which was located in the center of a given quadrant of the pool, was hidden 1 cm beneath the surface of water. Training was continuously conducted for 6 days (6 sessions), and every session consisted of 4 trials. In every trial, mouse was released from the wall of the tank by facing against to the wall into water and then was allowed to freely swim (search/find) in the pool, and to stand on the platform for 10 seconds within the 90-second testing period. An interval of 2 hours was set between two trials. In every training session, a starting quadrant and the order of quadrants from where mouse was put into water was randomly chosen so that both the starting quadrant and the order were different in different sessions in each animal, and was different between animals. Navigation was recorded by a videocamera, and the task performances including swimming paths, swimming speed, and time spent in each quadrant were recorded and analyzed by an EthoVision video tracking system (Noldus). A probe test was conducted 24 hours after the completion of the training. In this test, the platform was removed from the pool, and the task performances were recorded for 1 minute. The time spent in each quadrant was considered as the index for their memory retention.

### Novel object recognition test

Visual recognition memory was examined by using a novel object recognition test as described previously [64]. Briefly, mice were individually habituated in an open field (20×20×10 inches high) for 3 days (5 minutes×3 times per day). After this, training was conducted, in which two novel objects were placed into the open field, and mice were individually allowed to freely explore the box and objects for 8 minutes. Time spent on exploring each object was recorded. A retention test was conducted 24 hours after the training, and in this test, animals were individually placed back into the same box, in which one of the familiar objects used in the training session was replaced with a novel one, and were allowed to freely explore for 5 minutes. A preference index, e.g. the ratio of the amount of time spent on exploring any one of the two objects (training) or the novel one (retention test) over respective two objects was used as the index for the task performance.

### ELISA

ELISA was used to determine the serum level of both ACTH and CORT with commercially available kits (MD Bioproducts for ACTH; R&D systems for CORT). Experimental procedures followed the recommended steps described in the instruction of the kits. In order to have samples enough for triplicate measurements, blood was collected with a retroorbital eye bleeding method. In order to minimize non-specific effects, blood collection was conducted at 9:00 Am, and the procedure was completed within 30 seconds, by which time any possible change that might be produced by the sampling procedure was not yet measurable.

### Data analysis

Both female and male mice were mixed in each group. Given the role of genders in shifting fear memory and fear responses [65], a pre-statistical analysis between genders within group was conducted. With the exclusion of a significant gender effect, data were then analyzed with one-way or repeated ANOVA followed by *post-hoc* test such as Fisher's PLSD test, or with Student's *t* test. A p value that is less than 0.05 is considered significant.

## Supporting Information

Figure S1 **Transgene constructs for generation of transgenic mice.** (A). Expression cassette for CaMK-II-tTA transgenic mice. The tTA is flanked by an SV-40 intron/exon splicing signal (int) and an SV-40 ploy-A signal (pA). (B). Expression cassette for tetO-CCKR-2 transgenic mice. The CCKR-2 cDNA is flanked by an SV-40 intron/exon splicing signal (int) and a β-globin ploy-A signal (poly-A).
(DOC)
