# Supplementary material for: Bi-Directional Effect of Cholecystokinin Receptor-2 Overexpression on Stress-Triggered Fear Memory and Anxiety in the Mouse
Source: PLoS One. 2010 Dec 30;5(12):e15999. doi: 10.1371/journal.pone.0015999 (PMC3012733; doi:10.1371/journal.pone.0015999)
Supplement: Figure S1 — Transgene constructs for generation of transgenic mice. (A). Expression cassette for CaMK-II-tTA transgenic mice. The tTA is flanked by an SV-40 intron/exon splicing signal (int) and an SV-40 ploy-A signal (pA). (B). Expression cassette for tetO-CCKR-2 transgenic mice. The CCKR-2 cDNA is flanked by an SV-40 intron/exon splicing signal (int) and a β-globin ploy-A signal (poly-A). (DOC) [file pone.0015999.s001.doc]

8.5 kb 2.4 kb

0.4 0.3 1.3 kb 1.2 kb

*Xho I Sap I*

CCKR-2 cDNA

tetO

int

Poly-A

*Sal I Sal I*

A

B

    α-CaMKII promoter int tTA pA

**Figure S1.** Transgene constructs for generation of transgenic mice. (A). Expression cassette for CaMK-II-tTA transgenic mice. The tTA is flanked by an SV-40 intron/exon splicing signal (int) and an SV-40 ploy-A signal (pA). (B). Expression cassette for tetO-CCKR-2 transgenic mice. The CCKR-2 cDNA is flanked by an SV-40 intron/exon splicing signal (int) and a ß-globin ploy-A signal (poly-A).
